# Relationship Between the Gut Microbiome, Tryptophan-Derived Metabolites, and Osteoarthritis-Related Pain: A Systematic Review with Meta-Analysis

**DOI:** 10.3390/nu17020264

**Published:** 2025-01-12

**Authors:** Erika Meléndez-Oliva, Oliver Martínez-Pozas, Pierluigi Sinatti, Carmen Martín Carreras-Presas, Juan Nicolás Cuenca-Zaldívar, Silvia Turroni, Eleuterio A. Sánchez Romero

**Affiliations:** 1 Grupo de Investigación en Dietética Aplicada, Nutrición y Composición Corporal (DANuC), Department of Optics, Pharmacology and Anatomy, University of Alicante, 03690 Alicante, Spain; erika.melendez@ua.es; 2 Grupo de Investigación en Calidad de Vida y Salud, Departamento de Ciencias de la Salud, Universidad Europea de Valencia, 03016 Alicante, Spain; 3 Physiotherapy and Orofacial Pain Working Group, Sociedad Española de Disfunción Craneomandibular y Dolor Orofacial (SEDCYDO), 28009 Madrid, Spain; 4 Interdisciplinary Research Group on Musculoskeletal Disorders, Faculty of Sport Sciences, Universidad Europea de Madrid, 28670 Villaviciosa de Odón, Spain; plgsinatti@gmail.com; 5 Escuela Internacional de Doctorado, Faculty of Health Sciences, Universidad Rey Juan Carlos, 28922 Alcorcón, Spain; 6 IPPOCRATE Centro Medico Specialistico, Via La Spezia 38, 00055 Ladispoli, Italy; 7 Special Care Dentistry, Oral Medicine and Quality of Life Research Gorup (SOUL), Oral Medicine Unit, Faculty of Dentistry, European University of Madrid, 28670 Madrid, Spain; carmen.martin2@universidadeuropea.es; 8 Grupo de Investigación en Fisioterapia y Dolor, Departamento de Fisioterapia, Facultad de Enfermería y Fisioterapia, Universidad de Alcalá, 28801 Alcalá de Henares, Spain; 9 Research Group in Nursing and Health Care, Puerta de Hierro Health Research Institute-Segovia de Arana (IDIPHISA), 28222 Majadahonda, Spain; 10 Physical Therapy Unit, Primary Health Care Center «El Abajón», 28231 Las Rozas de Madrid, Spain; 11 Unit of Microbiome Science and Biotechnology, Department of Pharmacy and Biotechnology, University of Bologna, Via Belmeloro 6, 40126 Bologna, Italy; silvia.turroni@unibo.it; 12 Faculty of Medicine, Health and Sports, Department of Physiotherapy, Universidad Europea de Madrid, 28670 Villaviciosa de Odón, Spain

**Keywords:** osteoarthritis, gut microbiome, dysbiosis, inflammation, tryptophan

## Abstract

Introduction: Osteoarthritis (OA) is the most prevalent form of arthritis and affects over 528 million people worldwide. Degenerative joint disease involves cartilage degradation, subchondral bone remodeling, and synovial inflammation, leading to chronic pain, stiffness, and impaired joint function. Initially regarded as a “wear and tear” condition associated with aging and mechanical stress, OA is now recognized as a multifaceted disease influenced by systemic factors such as metabolic syndrome, obesity, and chronic low-grade inflammation. Recent studies have focused on the gut-joint axis to investigate how the gut microbiome modulates inflammation and pain in OA. Materials and Methods: A systematic review was conducted following the PRISMA guidelines and was registered with PROSPERO (CRD42024556265). This review included studies involving adults with symptomatic OA and analyzed the relationship between the gut microbiome and OA-related pain. Randomized and non-randomized clinical trials, case reports, editorials, and pilot studies were excluded. Searches were performed in PubMed, Cochrane Library, and Web of Science without publication date restrictions, and filtered for “observational studies”. The study selection and data extraction were performed by two independent researchers, and the risk of bias was assessed using appropriate tools. Results: Five observational studies were included in the systematic review, and three were included in the meta-analysis. Two studies reported an association between different tryptophan metabolites and pain levels in patients with OA. Two other studies demonstrated a correlation between lipopolysaccharide levels and pain in OA. A fifth study confirmed the relationship between *Streptococcus* relative abundance of *Streptococcus* spp. and knee pain. These results were not supported by a meta-analysis, which found no significant association between the presence of pain in OA and the presence of bacilli of the genus *Streptococcus* or plasma markers of the tryptophan pathway. Conclusions: Current evidence indicates a potential link between gut microbiome dysbiosis and OA-related pain. However, methodological limitations preclude definitive conclusions. Further research using advanced techniques and larger cohorts is needed to validate and extend these findings and elucidate the underlying mechanisms. Targeted manipulation of the gut microbiome may be a valuable strategy for pain management in OA patients.

## 1. Introduction

Osteoarthritis (OA) is the most prevalent form of arthritis and affects over 528 million people worldwide [1]. It poses significant healthcare challenges owing to the associated pain, functional limitations, and costs [2,3].

This degenerative joint disease is characterized by the breakdown of the articular cartilage, remodeling of the subchondral bone, formation of bone spurs (osteophytes), and inflammation of the synovium, leading to chronic pain, stiffness, and impaired joint function [4,5].

OA is a multifactorial disorder with many known risk factors including advanced age, obesity, family history, and joint overuse. OA is now understood to be a complex disease influenced by systemic factors, including metabolic syndrome, obesity, and chronic low-grade inflammation [4].

According to Leifer et al., OA imposes disability and pain on patients. As a consequence, patients experience a loss of quality of life and a worsening of their economic situation due to the direct and indirect costs of the disease. The authors concluded that there is a need to find an effective treatment with a multidisciplinary approach that can reduce the global economic cost of the disease [3].

Pain, a hallmark symptom of OA, is not only a consequence of joint damage but is also driven by systemic inflammatory processes [5]. Recent research has focused on the gut-joint axis, highlighting the role of the gut microbiome in modulating inflammation and pain in OA [6].

The gut microbiome is a diverse community of microorganisms (mainly bacteria, but also archaebacteria, fungi, and viruses) that resides in the gastrointestinal tract and influences host health through various mechanisms, including barrier effects against pathogen invasion, education and modulation of the immune system, and regulation of metabolism [7], possibly due to the multitude of bioactive molecules produced and/or contributed by gut microbes, which can enter the systemic circulation and exert effects even in distant organs [8]. Alterations in the composition of the gut microbiome, or dysbiosis, have been implicated in several chronic noncommunicable inflammatory diseases, including OA [9,10,11]. Dysbiosis may contribute to systemic inflammation and pain through the disruption of intestinal permeability (i.e., leaky gut) and production and/or stimulation of pro-inflammatory mediators [2,11,12,13]. The effects of the gut microbiome on energy and bone metabolism along the gut–brain axis have also been implicated [14]. Regarding the gut–brain axis, it has been suggested that dysbiosis contributes to central sensitization, a condition in which the nervous system becomes more sensitive to pain signals, leading to chronic pain [15,16].

The development and progression of OA are significantly influenced by inflammation, which is widely acknowledged as a key factor [17]. Among the most prevalent inflammatory mediators linked to this condition are the interleukins IL-6 and IL-1β, metalloproteinases (MMPs), and tumor necrosis factor α (TNF-α). Additionally, vascular endothelial growth factor plays a critical role in both initiating and advancing this inflammatory state [18,19,20].

Among the metabolites influenced by the gut microbiome, tryptophan (Trp)-derived compounds have gained attention for their roles in inflammation and pain modulation [10,20]. Trp is an essential amino acid that is the precursor of a range of microbial-derived metabolites in the indole, kynurenine, and serotonin pathways. The indole pathway produces ligands for the aryl hydrocarbon receptor (AhR), which plays a critical role in maintaining mucosal immunity and regulating gut permeability, thereby limiting the translocation of microbes and molecules [21]. The kynurenine pathway, mediated by the enzyme indoleamine 2,3-dioxygenase (IDO), is another Trp metabolic pathway associated with inflammation and neurotransmission [22,23,24]. Elevated levels of kynurenine and its metabolites have been linked to increased inflammation in OA, potentially contributing to the severity of the pain experienced by patients. The serotonin pathway, which is also derived from Trp, is involved in gut–brain communication and pain perception. Alterations in serotonin levels have been associated with increased pain sensitivity, further linking dysbiosis of the gut microbiome to OA-related pain [20]. Dysbiosis-induced alterations in Trp-derived metabolites may therefore contribute to OA in various ways, exacerbating systemic inflammation and pain [10,20].

Given the growing interest in the relationship between the gut microbiome and OA-related pain, this review aimed to explore how gut microbiome alterations may influence pain severity in OA. By examining the composition of the gut microbiome in individuals with symptomatic OA and assessing the association between specific microbial taxa, microbial products, and OA symptoms, including the inflammatory landscape, this review aims to uncover the potential mechanisms underlying the role of the gut microbiome in pain modulation. These findings may provide valuable insights for the development of microbiome-targeted interventions, such as dietary modifications, prebiotics, probiotics, synbiotics, and/or postbiotics, to manage pain in patients with OA.

## 2. Materials and Methods

This study was conducted following the Preferred Reporting Item for Systematic Reviews and Meta-Analysis (PRISMA) guidelines and was registered on PROSPERO prior to starting this review (CRD42024556265) [25]. The PRISMA Checklist is available in the Supplementary Material (Supplementary Material: PRISMA Checklist).

### 2.1. Inclusion Criteria

The study population included adults (aged 18 years and older) diagnosed with symptomatic OA (OA pain).

The review examined studies that compared different conditions related to the gut microbiome and OA pain, including the following:

Composition and function of the gut microbiome: Previous studies have investigated the relationship between the gut microbiome and OA-related pain, including assessments of microbial taxa and microbial products.

Outcomes: Primary outcomes included the severity of OA symptoms such as joint pain, swelling, and mobility issues. These studies evaluated the microbial profiles and inflammatory markers in OA patients to understand the association between gut microbiome alterations and the intensity of OA-related pain.

Measurements: Outcomes were assessed by profiling gut microbiome composition and function and examining inflammatory markers in relation to OA symptoms. Results are expressed mainly as means, standard deviations, and correlation co4efficients.

The analysis omitted duplicate publications, randomized and non-randomized clinical trials, case studies, editorial correspondence, preliminary investigations, opinion pieces, technical reports, research protocols, and literature reviews. The study incorporated articles written in any language.

### 2.2. Search Strategy

Articles were searched in PubMed, Cochrane Library Database, and Web of Science, without restrictions on publication date, up to July 2024. The search strategy combined medical subject headings (MeSH terms) and non-MeSH terms, adding a Boolean operator (OR and/or AND) to combine them. No language restrictions were imposed in accordance with clinical guidelines [26,27]. The search strategy was filtered by “observational studies”, which were incorporated into the study design.

The search utilized various MeSH and non-MeSH terms, including “osteoarthritis”, “gastrointestinal microbiome”, and “dysbiosis”. A comprehensive search strategy, detailing the specific search strings employed across all examined databases, is provided in the Supplementary Material. To identify potential additional studies, the reference lists of original research papers were manually examined. When necessary, the researchers reached out to authors for additional information.

### 2.3. Selection and Data Extraction

Two independent researchers (E.M.O. and P.S.) conducted a manual screening of all articles found in the database search. The initial review focused on titles and abstracts to determine potential eligibility based on inclusion criteria. Any challenges in screening were addressed by examining the full text. Subsequently, a comprehensive assessment was performed to confirm adherence to inclusion criteria. When disagreements arose regarding study eligibility, they were resolved through consensus or, if necessary, by consulting a third reviewer (E.A.S.R.). Data extraction was independently carried out by two researchers (E.M.O. and J.N.C.Z.). The information gathered from the included studies encompassed details about the author(s) and publication year, study objectives, research design, subject population, intervention methods, outcome measurements, and reported findings.

### 2.4. Risk of Bias Assessment

The ROBINS-I tool was employed to assess the potential for bias, examining areas such as confounding variables, participant selection, intervention classification, deviations from intended interventions, data gaps, outcome measurements, and selective reporting of results [28]. This tool is particularly suited for evaluating observational studies, such as cohort and case–control designs, where intervention groups are assigned during routine treatment decisions, and where randomization is not implemented.

Studies were categorized into five groups based on their risk of bias: “low”, “moderate”, “serious”, “critical”, or “no information available”. Two separate reviewers (O.M.P. and P.S.) independently evaluated the risk of bias in the chosen studies using identical methods, resolving any disagreements through discussion. The kappa coefficient (κ) was used to calculate inter-rater reliability, with κ > 0.7 indicating strong agreement between reviewers, κ of 0.5–0.7 suggesting moderate agreement, and κ < 0.5 denoting weak agreement [29].

### 2.5. Data Synthesis

Statistical analysis was performed using the R Ver. 4.1.3 program (R Foundation for Statistical Computing, Institute for Statistics and Mathematics, Welthandelsplatz 1, 1020 Vienna, Austria).

For the analysis, standardized ββ regression coefficients were reported as effect in the included studies. When the studies did not report coefficients or standard errors, they were calculated using appropriate formulas [30,31].

Due to the small number of articles included, a Bayesian meta-analysis with a random effect model [32,33] was performed using standardized ββ regression coefficients to specifically evaluate the association between OA-related pain and the presence of bacilli of the genus *Streptococcus* and markers of the Trp pathway in plasma.

Because some studies reported results adjusted for different baseline covariates, making their comparison difficult, the procedure described by Efthimiou et al. [34] was followed. Briefly, a sensitivity analysis was performed on the meta-analysis with all studies (naive pooling), assigning to the studies adjusted for different covariates a weight in their variance of 0.2, 0.5, and 0.8 and only with the studies adjusted for the same covariates, taking as a selection criterion the model with the most precise results in their confidence intervals and close to the model with only the studies adjusted for the same covariates. The selection of the a priori parameters of the models was performed using the method described by Ott et al. [35], by comparing the weakly informative distributions with the two reference posterior benchmarks based on the non-informative Jeffreys and Half-normal distributions and selecting the distribution that showed greater precision and was more informative than the reference ones.

Publication bias was assessed using the D measure based on the Copas Bayesian robust selection model, defined as negligible (<0.25), moderate (0.25–0.5), high (0.5–0.75) and very high (>0.75) [36].

## 3. Results

**Figure 1:**
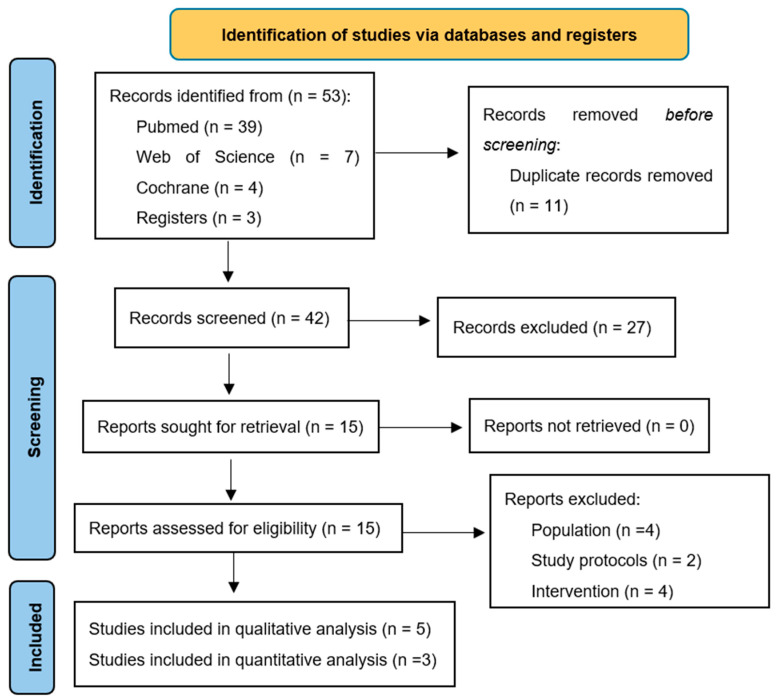
Prisma 2020 Flowchart.

Electronic database searches yielded 53 papers initially. Following the removal of duplicates and protocols, along with screening based on titles and abstracts, 15 papers were selected for comprehensive analysis. Subsequently, two researchers conducted a thorough review, resulting in the exclusion of ten studies (four carried out in mice, two research protocols, and four clinical trials with interventions) and the retention of five for qualitative analysis. Of these, three were ultimately included in the quantitative analysis. In accordance with PRISMA guidelines, a flowchart detailing the selection process and reasons for exclusion was developed and is presented in Figure 1.

### 3.1. Characteristics of the Included Studies

The present review included five studies, with a total of 756 adults diagnosed with OA, including 168 males and 588 females, with an average age of 66.1 years.

The main characteristics of the included studies are shown in Table 1.

### 3.2. Results of the Risk of Bias Assessment

**Figure 2:**
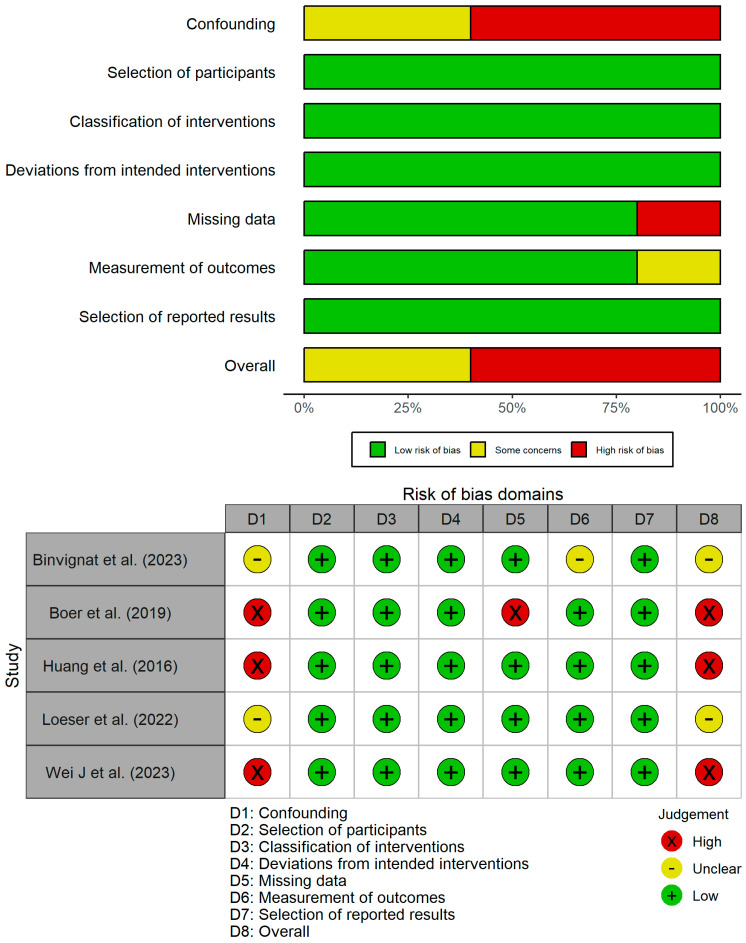
Risk of bias of non-randomized controlled trials (ROBINS-I) [13,14,16,20,37].

The results of the risk of bias assessment using the ROBINS-I tool are shown in Figure 2. Among the studies analyzed, two were identified as having a moderate risk of bias [13,20], while three as having a serious risk of bias [10,16,37]. The primary concerns pertained to the control of confounding variables, as several studies failed to account for potential influences, such as symptomatic slow-action drug osteoarthritis (SYSADOA) usage, hypertension, diabetes mellitus, smoking habits, or levels of physical activity. Additional biases were noted in relation to the measurement of the outcomes and instances of missing data. The inter-rater reliability was substantial, as indicated by a kappa coefficient (κ) of 0.615.

### 3.3. Data from Studies

#### 3.3.1. Relationship Between Alterations in Trp Metabolism and OA-Related Pain

Two of the included studies analyzed the association between Trp metabolites and pain in patients with OA.

Binvignat et al. [20] investigated the relationship between microbiota and Trp metabolism in hand OA (HOA) and found that 5-OH-Trp levels were positively correlated with the number of painful joints reported by patients. Furthermore, quinolonic acid and 3-OH kynurenine levels were positively correlated with pain as analyzed using the AUSCAN scale.

A study by Wei et al. [10] examined the connection between microbial function and crucial plasma metabolites associated with altered microbial activity in symptomatic hand osteoarthritis (SHOA). The researchers found that SHOA patients exhibited significantly modified microbial functions linked to tryptophan metabolism (Q = 0.025). These patients demonstrated elevated levels of 5-hydroxyindoleacetic acid (odds ratio [OR] = 1.25, 95% confidence interval [CI]: 1.09–1.42) and 5-hydroxytryptophol (OR = 1.13, 95% CI: 1.04–1.23) compared to individuals without SHOA. Conversely, SHOA patients showed reduced levels of indole-3-lactic acid (ILA) (OR = 0.85, 95% CI: 0.72–1.00), skatole (OR = 0.93, 95% CI: 0.88–0.99), and 3-hydroxyanthranilic acid (OR = 0.90, 95% CI: 0.85–0.96).

#### 3.3.2. Relationship Between Lipopolysaccharide (LPS) Levels and OA-Related Pain

Two of the included studies analyzed the association between LPS and pain in patients with OA.

A study by Huang et al. [37] examined the connection between lipopolysaccharide (LPS), an inflammatory microbial product, and various aspects of knee osteoarthritis (OA), including inflammation levels, symptoms, and radiographic changes. The research found that LPS and LPS-binding protein (LBP) in serum correlated with the presence of activated macrophages in the knee joint capsule (*p* = 0.01) and synovial membrane (*p* = 0.036). Similarly, LPS and LBP in synovial fluid (SF) were linked to activated macrophage abundance in the synovial membrane (*p* = 0.001 and *p* = 0.021, respectively). The severity of knee osteophytes was associated with serum LPS, LBP, and SF LPS levels (*p* = 0.030, *p* = 0.017, and *p* = 0.001, respectively). SF LPS showed a positive correlation with both knee joint space narrowing severity (*p* < 0.001) and total WOMAC score (*p* = 0.008). While serum LBPs tended to correlate with knee pain scores (*p* = 0.076), SF-LBP demonstrated a significant association (*p* = 0.039). The study revealed that LPS and LBP concentrations were notably lower in SF compared to serum (*p* < 0.0001). Additionally, serum LPS and LBP levels were strongly correlated (*p* < 0.001) and individually associated with body mass index (BMI) (*p* < 0.017) and plasma sCD14 levels (*p* < 0.001).

Loeser et al. [13] investigated whether a disrupted gut microbiome (dysbiosis) contributes to osteoarthritis (OA) associated with obesity. Their findings revealed no notable variations in alpha or beta diversity, or in genus-level composition between the study groups. Nevertheless, the subjects exhibited increased WOMAC pain scores and elevated plasma osteopontin (*p* = 0.01) and serum LPS (*p* < 0.0001) levels.

#### 3.3.3. Relationship Between Relative Abundance of Streptococcus and OA-Related Pain

In the Rotterdam Study, Boer et al. [16] examined the connection between joint discomfort, gut microbiome composition, and osteoarthritis-related knee pain. They discovered a significant link between microbiome beta diversity and knee WOMAC scores. The study revealed that individuals reporting higher pain levels on the WOMAC scale had an increased relative abundance of *Streptococcus* spp., independent of factors such as tobacco use, alcohol intake, or body mass index. A notable correlation was observed between the relative abundance of *Streptococcus* spp. and WOMAC knee pain scores. The association was strong (*p* = 1.4 × 10^−4^) and driven by local inflammation in the knee joint.

### 3.4. Quantitative Results: Meta-Analysis Description

It was confirmed that for both outcome variables (1) the presence of bacilli of the genus *Streptococcus* and (2) plasma markers of the tryptophan (Trp) pathway, the studies with the coefficients adjusted for the same covariates were those with the most precise CIs and were therefore selected (Supplementary Material: Figure S1). For both variables, the CIs in the Berger-Deely distribution were slightly wider than those in the DuMochel distribution (Supplementary Material: Table S1 and Supplementary Material: Figure S2). However, the a priori parameters showed that the Berger–Deely distribution was the most informative with a much smaller value of signed informativeness and a Hellinger distance closer to the anticonservative Jeffreys distribution than to the conservative half-normal distribution; therefore, the Berger–Deely distribution was selected (Supplementary Material, Table S1).

**Figure 3:**
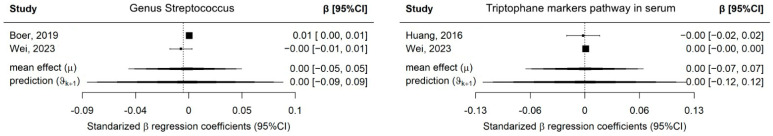
Outcome forest plots [14,16,37].

There was no significant association between the occurrence of pain in OA and the presence of bacilli of the genus *Streptococcus* (β = 0.003, 95% CI: −0.049–0.052, τ = 0.003, 95% CI: −0.049–0.052) or plasma Trp pathway markers (β = 0, 95% CI: −0.071–0.07, τ = 0, 95% CI: −0.071–0.07) (Figure 3).

In both models, the posterior distribution of the effect remained, with values close to zero as heterogeneity increased (Supplementary Material, Figure S3A) with a normal posterior distribution for this effect (Supplementary Material, Figure S3B), while in heterogeneity, it was not normal, with a tail on the right (Supplementary Material, Figure S3C). The distributions of both the effects and predictions were normal and coincident (Supplementary Material. Figure S4).

Both the model with the genus *Streptococcus* and the Trp pathway markers showed moderate publication bias (D = 0.351 and 0.296, respectively).

## 4. Discussion

To the best of our knowledge, this is the first systematic review with meta-analysis that analyzes the relationship between gut microbiota and pain in patients with OA. The results of the observational studies analyzed individually suggest that pain in OA is associated with alterations in Trp metabolism and the gut microbiome, namely the relative abundance of a specific genus, *Streptococcus*, microbial functions related to Trp metabolism, and LPS levels. Nevertheless, these results were not supported by the meta-analysis, most likely due to its limitation in the number of studies included. Binvignat et al. [20] found that elevated levels of 5-OH-Trp, quinolinic acid, and 3-OH kynurenine correlated with an increase in the number of painful joints, suggesting that these metabolites may enhance neuroinflammation, and therefore, pain. Interestingly, Bingvignat et al. [20] reported a positive correlation between circulating levels of 5-OH Trp and the number of painful joints in patients, a finding that contrasts with the known analgesic effects of 5-OH Trp supplements in conditions such as fibromyalgia. This apparent paradox may reflect differences in the metabolic context, receptor sensitivities, or inflammatory milieu influencing endogenous versus exogenous 5-OH Trp activity. These findings underscore the complexity of tryptophan metabolism and its impact on pain modulation, highlighting the need for further research to elucidate these mechanisms.

Wei et al. [20] identified a more complex pattern, with elevated levels of 5-hydroxyindoleacetic acid and 5-hydroxytryptophol and decreased levels of metabolites, such as ILA and skatole, suggesting impaired microbial function related to Trp metabolism and an imbalance of Trp metabolites that could affect pain perception. Both studies agreed that Trp metabolism plays a crucial role in the pathogenesis of pain; however, Binvignat et al. [20] focused on the direct correlation between specific metabolites and pain. They highlighted that an altered microbial function is associated with SHOA.

However, the association between LPS levels and pain in OA reinforces the importance of inflammatory factors in this disease. Huang et al. [20] found that LPS and LBP levels correlated with inflammation and structural changes in the knee joint, such as narrowing of the joint space and presence of osteophytes. These findings suggest that LPS acts as a local mediator of inflammation in joints, thereby amplifying pain. Additionally, Loeser et al. [20] found that elevated serum LPS and osteopontin levels are associated with increased pain in obesity-linked OA, underscoring the impact of systemic inflammation on disease progression. Although they found no significant differences in the diversity and genus-level composition of the gut microbiome between cases and controls, their study reinforces the idea that microbial components, such as LPS, may act both locally and systemically to increase pain.

Finally, Boer et al. [20] provided evidence that a higher relative abundance of *Streptococcus* spp. in the gut microbiome is significantly associated with greater pain in knee OA, independent of factors such as tobacco use, alcohol consumption, and BMI. This association implies that some microbial components may directly influence local inflammation and exacerbate pain in affected joints.

Taken together, these studies suggest that some members of the gut microbiome, structural components, and products derived from their metabolism may play a critical role in the onset and severity of pain in OA, both through local inflammatory processes and modulation of systemic inflammation. Furthermore, obesity appeared to amplify these effects. These findings may pave the way for the development of precise personalized treatments based on modulation of the gut microbiome, including *Streptococcus*, Trp metabolism, and LPS, to reduce pain and improve the quality of life in patients with OA.

Recent studies support the importance of dietary interventions as a complementary therapeutic approach in the management of OA [38,39]. Diet plays a critical role in modulating the gut microbiome, which in turn has a significant impact on the pathophysiology of OA. Microbiome-derived metabolites, such as short-chain fatty acids (SCFAs), can directly influence osteoclast activity, suppressing their differentiation and, therefore, preventing bone loss. Furthermore, dietary components such as prebiotics promote the growth of beneficial bacteria that generate anti-inflammatory metabolites and regulate the immune balance, such as Th17 and Treg cells, crucial in the inflammatory progression of OA [39,40]. On the other hand, an unbalanced diet, high in saturated fats and refined sugars, can alter microbiome diversity and promote chronic inflammation, exacerbating OA symptoms [39].

### Limitations a Future Directions

We are aware that our study has certain limitations. The small number of studies considered and the heterogeneity among them made it difficult to perform a robust meta-analysis that supported the individual findings. Likewise, the small sample sizes partially limit the firmness of the conclusions obtained. In the future, it will be essential to carry out more observational research with large population samples that explore in detail the relationship between the microbiota and pain in OA, in order to confirm these preliminary results.

Additionally, while diet, including protein and tryptophan intake, is a recognized modulator of gut microbiota composition and function, this review did not explore dietary aspects in detail. Future studies should investigate the role of tryptophan-rich dietary patterns in shaping gut microbiota and its metabolites, particularly in the context of osteoarthritis-related pain.

## 5. Conclusions

Pain related to OA appears to be closely linked to metabolic alterations (especially Trp metabolites) and microbial factors such as the presence of LPS and certain gut bacteria (*Streptococcus* spp.). Local inflammation and metabolic changes may be key determinants of the intensity of pain experienced by patients. A larger number of studies with high methodological quality (including higher taxonomic and functional resolution for gut microbiome profiling and untargeted metabolomics) are needed to enable a robust and insightful meta-analysis of results.

## Figures and Tables

**Table 1:** Characteristics of the included studies. For each study, the aim, study design, participants, type of intervention, outcome measures and main results are reported.

Author(s) (Year)	Aim of the Study	Study Design	Participants	Outcome Measures	Reported Resutls
Binvignat et al. [20]	To investigate host and gut microbiota related Trp metabolism in hand osteoarthritis (HOA)	A cross-sectional study (observational cohort)	416 HOA: erosive (*n* = 141) and non-erosive HOA (*n* = 275). Final cohort consisted of 84% female, mean age 66.7 (±7.2) years, mean BMI 25.1 (±4.3) kg/m^2^, and mean Australian Canadian osteoarthritis hand index (AUSCAN) pain score 25.8 (±21.4)	The study assessed serum levels of Trp metabolites, their ratios, and metabolic pathway activations, including compounds from the kynurenine-IDO pathway (3H-KYN, kynurenic acid, xanthurenic acid, 3-HAA, picolinic acid, quinolinic acid), serotonin pathway (5-HTP, serotonin, 5-hydroxyindoleacetic acid, N-acetyl-serotonin, melatonin), and indole-AHR derivatives (indole 3-lactic acid, indole-3-acetamide, Iald, indole-3-acetic acid, and indoxyl sulfate), as well as tryptamine and tryptophol. CRP, patient-reported painful and tender joints, AUSCAN subscores (pain, physical function, and stiffness), HADS, and AIMS2-SF components (symptoms, affective, and social) were also measured.	The study discovered correlations between erosive HOA and various serum Trp metabolites, ratios, and a metabolic pathway. Significant associations were found for Iald (*p* = 0.026), 3-HAA (*p* = 0.004), 5-HTP (*p* = 0.014), tryptophan (*p* = 0.007), and several metabolite ratios (*p* < 0.05) (indoxyl sulfate/tryptophan, quinolonic acid/3-HAA, xanthurenic acid/3H-KYN, 3-HAA/3H-KYN, 3H-KYN/kynurenine, 5-HIAA/5-HTTP, and 5-HTP/tryptophan). The kynurenine-IDO pathway also showed a significant association (*p* = 0.04). Furthermore, eleven metabolites correlated with HOA symptoms, primarily pain, with patient-reported painful joints positively correlating with 5-OH-Trp levels (r = 0.26, *p* < 0.0019), and AUSCAN pain scores positively correlating with Quinolinic acid (r = 0.16, *p* = 0.006) and 3-OH kynurenine (r = 0.14, *p* = 0.02).
Wei et al. [10]	To assess the relationship between microbial function and key plasma metabolites related to altered microbial function in symptomatic HOA (SHOA)	Observational study (discovery cohort and validation cohort)	Discovery cohort, *n* = 1359 (70 participants with SHOA, of which 59.2% female, age 72.0 ± 6.0 years); Validation cohort, *n* = 142 (71 with SHOA, of which 59.2% female, age 70.6 ± 8.2 years)	Stool collection, DNA extraction, DNA library construction, shotgun metagenomic sequencing, and plasma and Trp metabolite assessment	Patients with SHOA showed significantly altered Trp metabolism (Q = 0.025), with increased levels of 5-hydroxyindoleacetic acid (OR = 1.25, 95% CI: 1.09–1.42) and 5-hydroxytryptophol (OR = 1.13, 95% CI: 1.04–1.23), but decreased levels of indole-3-lactic acid (ILA) (OR = 0.85, 95% CI: 0.72–1.00), skatole (OR = 0.93, 95% CI: 0.88–0.99), and 3-hydroxyanthranilic acid (OR = 0.90, 95% CI: 0.85–0.96). The validation cohort confirmed lower ILA levels were associated with SHOA (OR = 0.70, 95% CI: 0.53–0.92).
Loeser et al. [13]	To test the hypothesis that altered gut microbiota (dysbiosis) plays a role inobesity-associated OA	Observational study (case–control)	OA cases (*n* = 50) had hand plus knee OA (Kellgren–Lawrence [KL] grade ≥2 or arthroplasty), age 73.7 ± 6.9 years, 86% female; Controls (*n* = 42) had no hand OA and KL grade 0–1 knees, 70.8 ± 6.4 years, 62% female.	Stool and blood samples from 92 obese participants (BMI ≥ 30 kg/m^2^) of the Johnston County Osteoarthritis Project were analyzed. 16S rRNA amplicon sequencing determined alpha and beta diversity and taxa abundance differences in stool samples. Multiplex cytokines, lipopolysaccharide (LPS), and LPS-binding protein (LBP) levels were measured in blood samples. Germ-free mice, gavaged with case or control pooled fecal samples, were fed a high-fat, high-sucrose diet for 40 weeks, and their knee OA was histologically evaluated.	OA cases were slightly older with more females and higher BMI, WOMAC pain, and KL grades than controls. There were no significant differences in alpha or beta diversity or genus-level composition between cases and controls. Cases had higher plasma levels of osteopontin (*p* = 0.01) and serum LPS (*p* < 0.0001). Mice transplanted with case or control microbiota exhibited a significant difference in alpha diversity (*p* = 0.02) and beta diversity, but no differences in OA severity.
Boer et al. [16]	The Rotterdam Study aimed to examine the connection between gastrointestinal microbiome composition and joint pain, specifically focusing on knee pain associated with osteoarthritis (OA)	Observational study (case–control)	1427 patients (821 women), mean age 56.8 ( ± 5.9) years.Inclusion criteria: patients with knee OA(cases) and without knee OA (controls) fromthe Rotterdam Study	16S rRNA gene-based Illumina sequencingfor microbiome profiling; WOMAC index	Results indicated a significant link between microbiome beta diversity and knee WOMAC scores. Subjects reporting higher WOMAC pain had increased Streptococcus abundance, regardless of tobacco use, alcohol consumption, and BMI. The relative abundance of Streptococcus spp. strongly correlated with WOMAC knee pain scores (*p* = 1.4 × 10^−4^), attributed to localized knee joint inflammation.
Huang et al. [37]	This study aimed to examine the connection between lipopolysaccharide (LPS), a microbial pro-inflammatory agent, and inflammation levels, symptoms, and radiographic changes in knee osteoarthritis (OA)	Observational study (cohort study)	The study included 25 participants (18 females) from the Etarfolatide cohort, with an average age of 62.41 (±15.8) years. Subjects were required to have radiographic knee OA (unilateral or bilateral [K/L] grade 1–4) for inclusion	LPS was quantified using the EndoZyme assay, which was carefully optimized for systemic and synovial fluid (SF) analyses. Lipopolysaccharide-binding protein (LBP) was evaluated in both serum and SF to determine its relationship with OA phenotypic outcomes, using a commercial sandwich ELISA kit. Statistical models were adjusted for age, gender, and BMI. The WOMAC index was utilized for assessment.	Serum LPS and LBP correlated with activated macrophages in the knee joint capsule (*p* = 0.01) and synovium (*p* = 0.036), while SF LPS and LBP were linked to synovial activated macrophages (*p* = 0.001 and *p* = 0.021). Serum LPS, LBP, and SF LPS were associated with knee osteophyte severity (*p* = 0.030, *p* = 0.017, and *p* = 0.001), and SF LPS correlated with joint space narrowing severity (*p* < 0.001) and total WOMAC score (*p* = 0.008). SF LBP significantly correlated with self-reported knee pain score (*p* = 0.039), while serum LBP showed a trend towards a positive association (*p* = 0.076). LPS and LBP concentrations were significantly lower in SF than in serum (*p* < 0.0001), and serum LPS and LBP levels strongly correlated (*p* < 0.001) and were associated with BMI (*p* < 0.017) and plasma sCD14 (*p* < 0.001).

AHR: Aryl Hydrocarbon Receptor; BMI: Body mass index; BP: Blood pressure; CRP: C-reactive protein; EA: Electroacupuncture; GLM: Green-lipped mussel extract; 3-HAA: 3-OH anthranilic acid; HADS: Hospital Anxiety Depression Scale; 5-HIAA: 5-Hydroxyindoleacetic acid; 3H-KYN: 3-Hidroxykynurenine; 5-HTP: 5-hydroxytryptophan; IDO: Indoleamine 2,3-dioxygenase; ILA: indole-3-lactic acid; K/L: Kellgreen/Lawrence grading system; LBP: LPS-binding protein; LPS: Lipopolysaccharide; NRS: Numerical rating scale; OA: Osteoarthritis; OR: Odds ratio; *p*: *p*-value; sCD14: Soluble cluster of differentiation 14; SF: Synovial fluid; SHOA: Symptomatic hand osteoarthritis; Trp: Tryptophan; WOMAC: Western Ontario and McMaster Universities Osteoarthritis Index.

## Data Availability

The data presented in this study are available on request from the corresponding authors.

## Supplementary Materials

The following supporting information can be downloaded at: https://www.mdpi.com/article/10.3390/nu17020264/s1, Figure S1: Equal vs. unequal covariates adjusted for sensitivity analysis; Figure S2: Reference prior analysis accuracy; Figure S3: Posterior distribution plots; Figure S4: Posterior effect distribution vs. prediction plots; Table S1: Reference prior analysis. Reference [41] are cited in the Supplementary Materials.
