# Supplementary material for: Relationship Between the Gut Microbiome, Tryptophan-Derived Metabolites, and Osteoarthritis-Related Pain: A Systematic Review with Meta-Analysis
Source: Nutrients. 2025 Jan 12;17(2):264. doi: 10.3390/nu17020264 (PMC11767305; doi:10.3390/nu17020264)
Supplement: Supplementary file 1 [file nutrients-17-00264-s001.zip › nutrients-3373686-supplementary.pdf]

Supplementary material

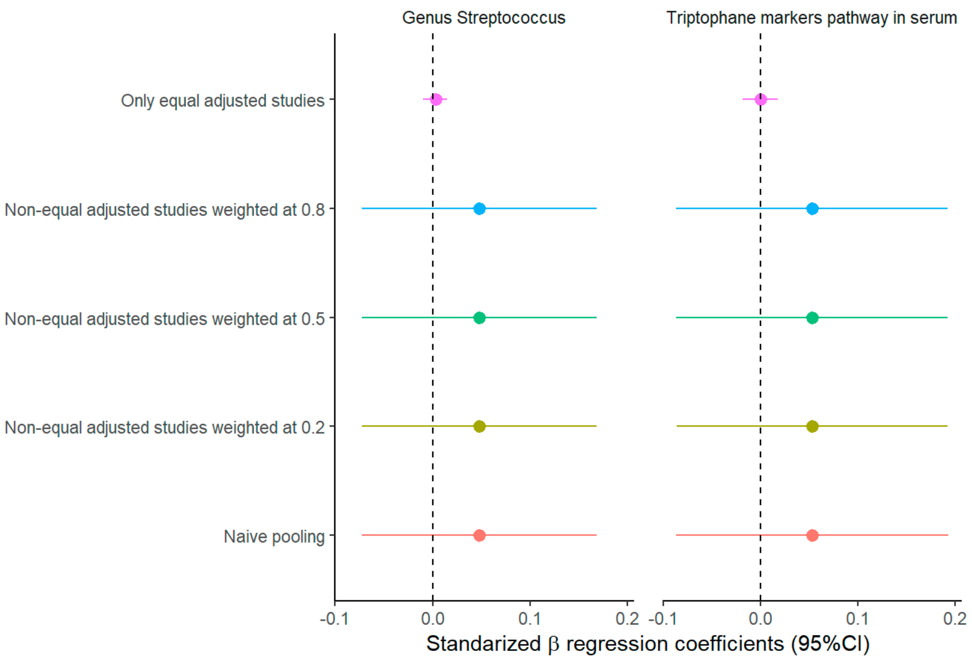

Supplementary material. Figure S1: Equal vs. unequal covariates adjusted for sensitivity analysis.

Supplementary material. Table S1: Reference prior analysis.

	Genus *Streptococcus*				Trp markers pathway in serum		
**Prior parameter**	**Prior distribution**	**95%CI amplitude**	**Hellinger distance**	**Signed informativeness**	**95%CI amplitude**	**Hellinger distance**	**Signed informativeness**
Effect (μ)	Berger-Deely	0.1	0.454	0.07	0.137	0.483	0.087
	DuMouchel	0.015	0.131	0.284	0.02	0.159	0.256
	Half-Cauchy	1.279	0.697	0.4	1.27	0.674	0.365
	Half-normal	*	*0*	*	*	*0*	*
	Jeffreys	*	*0.388*	*	*	*0.401*	*
	Uniform	4.788	0.745	0.496	4.767	0.727	0.469
Heterogeneity (τ)	Berger-Deely	0.174	0.659	0.119	0.238	0.731	0.141
	DuMouchel	0.015	0.274	0.403	0.019	0.298	0.391
	Half-Cauchy	1.636	0.83	0.462	1.628	0.817	0.426
	Half-normal	*	*0*	*	*	*0*	*
	Jeffreys	*	*0.559*	*	*	*0.613*	*
	Uniform	7.16	0.855	0.549	7.144	0.844	0.521
Prediction (Θ)	Berger-Deely	0.172	0.472	0.074	0.237	0.517	0.093
	DuMouchel	0.024	0.141	0.296	0.03	0.174	0.272
	Half-Cauchy	2.247	0.71	0.403	2.23	0.695	0.369
	Half-normal	*	*0*	*	*	*0*	*
	Jeffreys	*	*0.403*	*	*	*0.43*	*
	Uniform	9.934	0.756	0.499	9.883	0.744	0.472

Green:Posterior benchmark reference distributions.

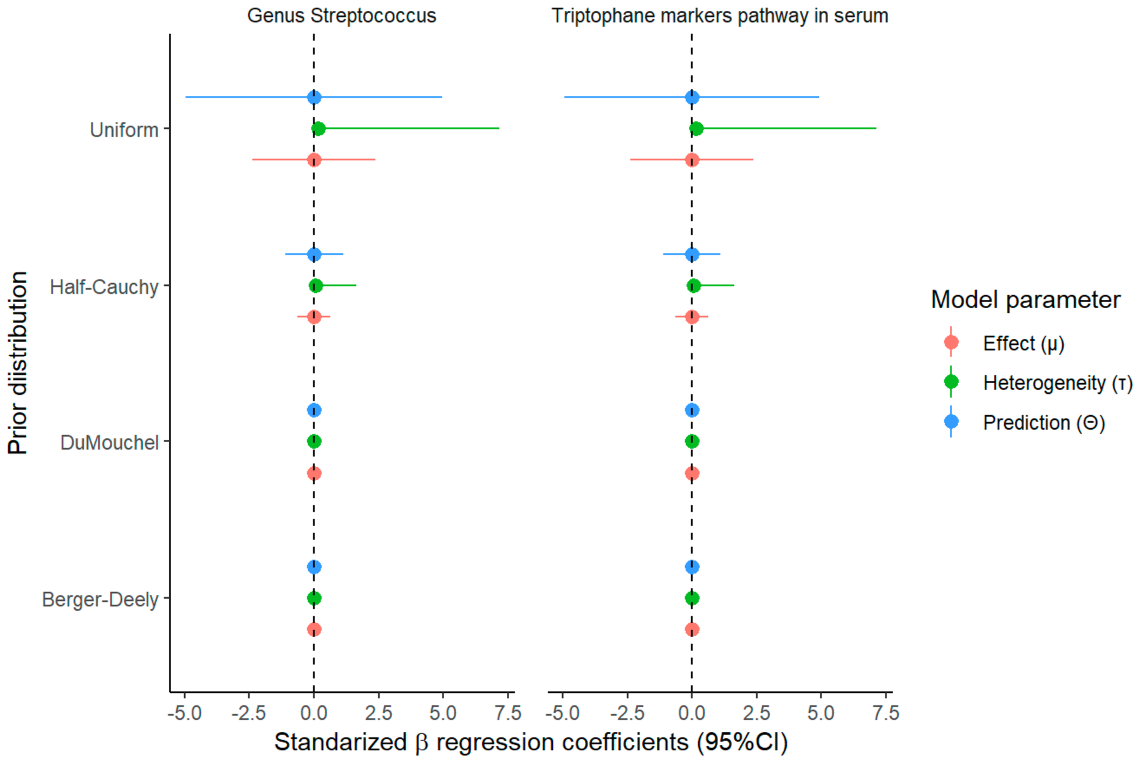

Supplementary material. Figure S2: Reference prior analysis accuracy.

Genus Streptococcus

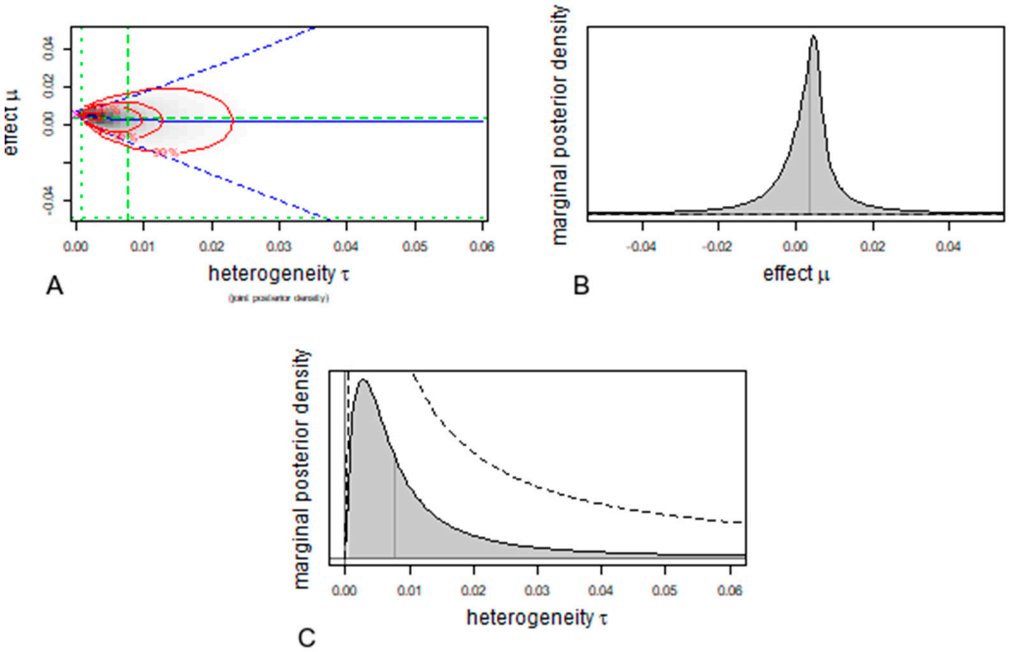

Triptophane markers pathway in serum

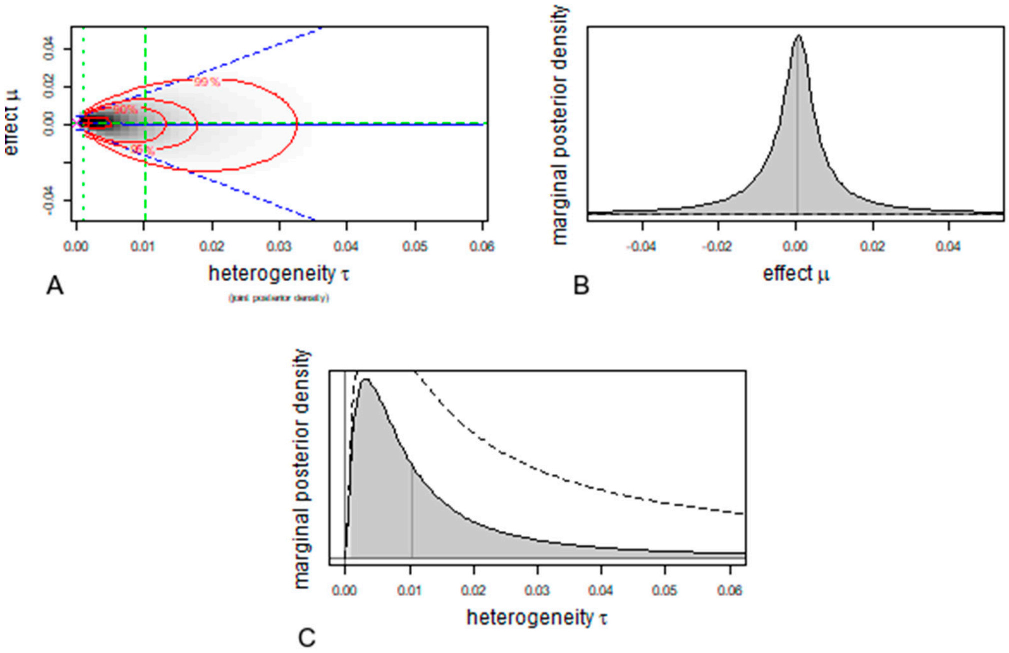

Supplementary material. Figure S3: Posterior distribution plots.

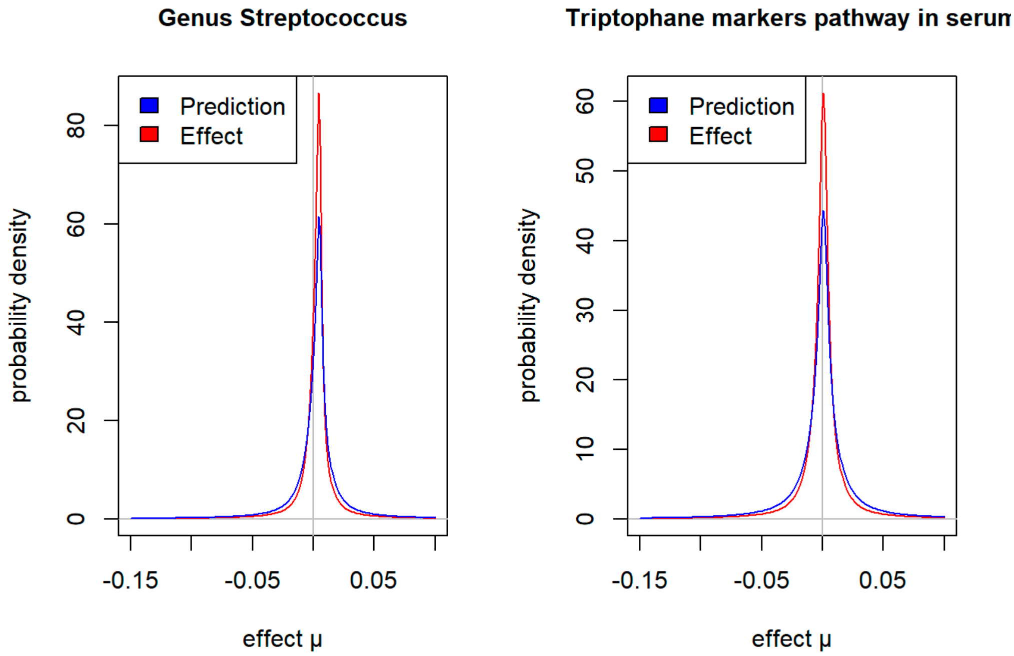

Supplementary material. Figure S4: Posterior effect distribution vs. prediction plots
